# Bioinformatics Research in Bacterial Genomics and Metagenomics

**DOI:** 10.3390/cimb47040258

**Published:** 2025-04-08

**Authors:** Vesselin Baev

**Affiliations:** Department of Molecular Biology, Faculty of Biology, University of Plovdiv, Tzar Assen 24, 4000 Plovdiv, Bulgaria; baev@uni-plovdiv.bg

Bioinformatics plays a crucial role in bacterial genomics and metagenomics research, transforming our understanding of microbial communities and their functions. The rapid advancement of DNA sequencing technologies has resulted in an explosion of genomic data, necessitating sophisticated computational tools for analysis [1]. Moreover, computational tools enable the efficient processing and interpretation of large-scale genomic datasets, facilitating the identification of gene functions, metabolic pathways, and regulatory networks in bacteria.

In the realm of next-generation sequencing and long-read sequencing technologies, Oxford Nanopore Technologies (ONT) have had a particularly revolutionizing role in bacterial genomics by enabling complete genome assemblies and resolving complex structural elements. Nanopore sequencing provides ultra-long reads that span repetitive regions and plasmids, overcoming the limitations of short-read technologies like Illumina [2]. Recent advancements in ONT chemistry, such as the R10.4.1 flow cells, have significantly improved read accuracy, allowing standalone long-read assemblies to replace hybrid approaches. This eliminates the need for costly short-read validation and enables real-time analysis [3]. Additionally, the portability and compact size of ONT hardware facilitate its use in small-scale laboratory environments and even in field studies.

Long-read assemblers generate high-quality complete bacterial genomes that can be further easily annotated with various bioinformatics tools [4]. This accelerates the exploration of new bacterial strains and enhances our ability to study antimicrobial resistance (AMR) profiles and virulence factors, which is crucial for evaluating bacterial pathogenic potential [5,6,7]. Furthermore, ONT’s portability facilitates rapid pathogen detection in clinical settings, which can be further complemented by full-length 16S rRNA sequencing to enhance microbial community analysis at a deeper resolution (Figure 1).

In metagenomics, bioinformatics tools are essential for annotating sequence data from complex microbial communities. Whether using traditional 16S rRNA amplicon sequencing or shotgun sequencing approaches, these tools allow researchers to explore microbial diversity and investigate microbe–environment interactions. Developing new tools for metagenomics exploration and data visualization is a key step in advancing the field [8]. Shotgun metagenomic sequencing, in particular, offers several advantages over 16S rRNA sequencing [9]. It provides a more comprehensive view of microbial communities by capturing the full genetic content of all organisms present, including bacteria, archaea, and viruses. This technology enables higher taxonomic resolution—often at the species or even strain level—compared to the genus-level resolution typically achieved by 16S rRNA sequencing. Additionally, shotgun metagenomics reveals bacterial functional repertoires by identifying genes and metabolic pathways, providing insights into microbial capabilities beyond mere taxonomic classification. It also avoids the PCR-related biases associated with 16S rRNA amplicon sequencing, resulting in a more accurate representation of community composition. While traditionally more expensive, recent progress in shallow shotgun sequencing has made it more cost-competitive with 16S rRNA sequencing, while still providing superior taxonomic and functional insights [10].

The integration of various ’omics’ data through bioinformatics approaches offers a more comprehensive understanding of bacterial physiology and ecology [11]. This integration is particularly valuable in fields such as environmental science and biotechnology, where microbial strains are optimized for enzyme production or bioremediation. Furthermore, bacterial comparative genomics enables researchers to analyze and compare hundreds of bacterial genomes simultaneously. This bioinformatics approach has led to significant insights into bacterial evolution, adaptation, and the development of new therapeutic strategies against pathogens. By leveraging comparative genomics, researchers can uncover patterns of gene conservation and divergence, assisting in the identification of novel drug targets and vaccine candidates [12].

In summary, bioinformatics is at the forefront of bacterial genomics and metagenomics, providing powerful tools to analyze, interpret, and apply genomic data in various scientific and medical fields. With continuous advancements in sequencing technologies and computational methods, our ability to study microbial communities will continue to expand, driving innovations in medicine, biotechnology, and environmental science.

## Figures and Tables

**Figure 1:**
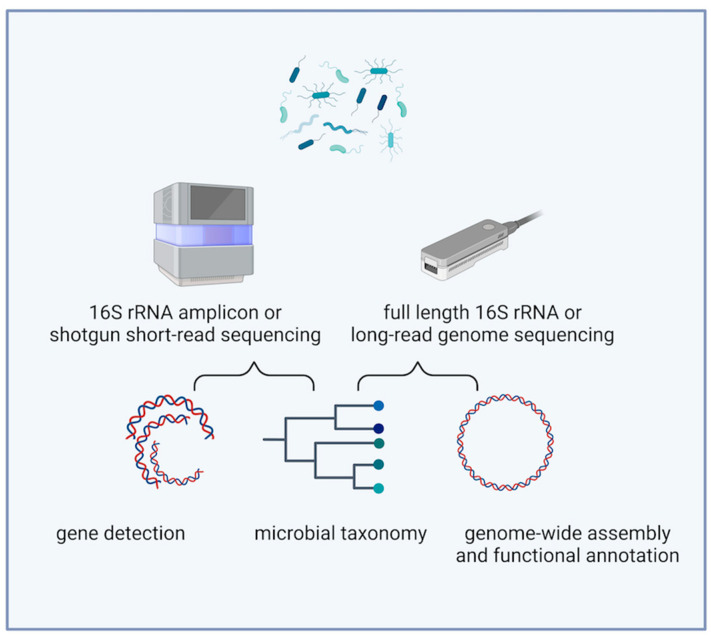
Next-generation sequencing methods for exploring bacterial genomics and metagenomics (Created with BioRender.com).
